# Plasma Ascorbic Acid, *A Priori* Diet Quality Score, and Incident Hypertension: A Prospective Cohort Study

**DOI:** 10.1371/journal.pone.0144920

**Published:** 2015-12-18

**Authors:** Brian Buijsse, David R. Jacobs, Lyn M. Steffen, Daan Kromhout, Myron D. Gross

**Affiliations:** 1 Division of Epidemiology and Community Health, School of Public Health, University of Minnesota, Minneapolis, Minnesota, United States of America; 2 Department of Epidemiology, German Institute of Human Nutrition Potsdam-Rehbruecke, Nuthetal, Germany; 3 Division of Human Nutrition, Wageningen University, Wageningen, the Netherlands; 4 Department of Laboratory Medicine and Pathology, University of Minnesota, Minneapolis, Minnesota, United States of America; University of Nebraska Medical Center, UNITED STATES

## Abstract

Vitamin C may reduce risk of hypertension, either in itself or by marking a healthy diet pattern. We assessed whether plasma ascorbic acid and the *a priori* diet quality score relate to incident hypertension and whether they explain each other’s predictive abilities. Data were from 2884 black and white adults (43% black, mean age 35 years) initially hypertension-free in the Coronary Artery Risk Development in Young Adults Study (study year 10, 1995–1996). Plasma ascorbic acid was assessed at year 10 and the diet quality score at year 7. Eight-hundred-and-forty cases of hypertension were documented between years 10 and 25. After multiple adjustments, each 12-point (1 SD) higher diet quality score at year 7 related to mean 3.7 μmol/L (95% CI 2.9 to 4.6) higher plasma ascorbic acid at year 10. In separate multiple-adjusted Cox regression models, the hazard ratio of hypertension per 19.6-μmol/L (1 SD) higher ascorbic acid was 0.85 (95% CI 0.79–0.92) and per 12-points higher diet score 0.86 (95% CI 0.79–0.94). These hazard ratios changed little with mutual adjustment of ascorbic acid and diet quality score for each other, or when adjusted for anthropometric variables, diabetes, and systolic blood pressure at year 10. Intake of dietary vitamin C and several food groups high in vitamin C content were inversely related to hypertension, whereas supplemental vitamin C was not. In conclusion, plasma ascorbic acid and the *a priori* diet quality score independently predict hypertension. This suggests that hypertension risk is reduced by improving overall diet quality and/or vitamin C status. The inverse association seen for dietary but not for supplemental vitamin C suggests that vitamin C status is preferably improved by eating foods rich in vitamin C, in addition to not smoking and other dietary habits that prevent ascorbic acid from depletion.

## Introduction

The favorable effects of vitamin C on blood pressure have been researched for a long time. In a meta-analysis of 29 short-term (<1 y) and mostly small-sized randomized controlled trials [1], vitamin C supplementation was found to moderately lower blood pressure. Even though the magnitude of effect differed across the trials, the favorable effect was seen for vitamin C dosages as low as 60 mg/day. The effect of vitamin C on blood pressure may be related to the improvement in synthesis of and bioavailability in nitric oxide [2].

If vitamin C has a clinically meaningful favorable effect on blood pressure, this may translate to a lower rate of cardiovascular diseases. Randomized controlled trials of vitamin C supplementation have, however, failed to show a reduction in the incidence of cardiovascular diseases [3]. Although the design of some of the trials may be criticized [4], their null results also raise questions about the possibility of uncontrolled confounding in observational studies that reported inverse associations of plasma ascorbic acid and cardiovascular disease risk [5–9]. For example, vitamin C may lower blood pressure not in itself, but rather by marking high intakes of fruit and vegetables [10] or plant foods [11], which are rich in a multitude of nutrients with potential health benefits [12–14], or of diet pattern [15, 16].

Only one prospective study reported on plasma ascorbic acid and change in blood pressure over time [17]. Although that study reported an inverse association, the investigators were not able to evaluate whether this is due to better diet quality in persons with higher vitamin C status. Therefore, in the current study, we assessed whether circulating concentrations of ascorbic acid and a previously constructed a priori diet quality score relate to incident hypertension in a cohort of community-dwelling middle-aged US adults. We hypothesized that both plasma ascorbic acid and the a priori diet quality score are inversely associated with incident hypertension. Because plasma ascorbic acid and the a priori diet quality score represent similar phenomena, we also hypothesized that each would explain a portion of the predictive capability of the other.

## Materials and Methods

### Study population

CARDIA is a prospective cohort study on the evolution of risk of coronary disease. Briefly, 5,115 black and white participants aged 18–30 years were recruited in 1985–1986 (year 0) [18] in 4 US metropolitan areas (Birmingham, AL; Chicago, IL; Minneapolis, MN; and Oakland, CA). Participants were re-examined after 2, 5, 7, 10, 15, 20, and 25 years, with participation rates among surviving cohort members of 91%, 86%, 81%, 79%, 74%, 72%, and 72% respectively. Recruitment was designed to include approximately equal numbers of participants at each clinical site by age (45% aged 18–24 years, 55% aged 25–30 years), race (52% black, 48% white), sex (46% men, 54% women), and education (40% ≤12 years of education, 60% >12 years). Institutional review board approval and written informed consent were obtained at each site at every examination.

Plasma ascorbic acid was measured for the first time at CARDIA year 10. The nearest assessment of diet was at year 7. Because the a priori diet score used in the current study shows strong tracking correlation within participants over time [19], its unmeasured year 10 value is likely close to its observed year 7 value. We investigated changes in blood pressure between year 10 and year 25 for both plasma ascorbic acid and a priori diet score.

At year 10, 3950 individuals were examined, 3643 of whom provided a fasting blood sample (92.2%). Plasma ascorbic acid was measured in 3564 people. For the analysis on the association between plasma ascorbic acid and incident hypertension, we further excluded observations for the following reasons: history of hypertension up to and including year 10 (n = 277); missing blood pressure or adjustment variable values at year 10 (n = 227); and no follow-up examination after year 10 (n = 176). Accordingly, 2884 people were included in this analysis. For the association between the a priori diet quality score at year 7 and incident hypertension between year 10 and 25, we further excluded those with missing values of diet intake, yielding a sample of 2596 people. Blood pressure values were on average similar between the 2596 people with information on year 7 dietary intake and the 288 without (P value unpaired t-test for systolic and diastolic blood pressure 0.10 and 0.14, respectively).

### Ethics

All participants provided written informed consent at each exam, and institutional review boards from each field center and the coordinating center (The University of Alabama Birmingham Institutional Review Board, University of Minnesota Institutional Review Board, Kaiser Permanente Northern California Institutional Review Board) provided approval for the study.

### Blood collection and measurement of blood concentrations of ascorbic acid

Fasting venous blood was sampled at years 10 and 15. Plasma ascorbic acid was measured as part of an ancillary study to CARDIA by the Molecular Epidemiology and Biomarker Laboratory (University of Minnesota), by high-performance liquid chromatography (HPLC). Metaphosphoric acid was added to the plasma samples to stabilize ascorbic acid concentrations during storage at –70°C for up to one year. Quality-control procedures involved adding high and low control pools to the plates, with coefficients of variation being between 5 and 8%.

### Dietary assessment and *a priori* diet quality score

Usual diet was assessed at year 7 (1992–1993) using an interviewer-administered dietary history method [20]. Interviewers asked open-ended questions about food consumption in the past month, including food item, additions at the table and fat used in food preparation, frequency, and portion size. Food intake was first categorized into 166 food groups, then collapsed into 46 food groups. The diet quality score was defined a priori by the investigators’ ratings of these food groups as beneficial (20 groups), adverse (13 groups), or neutral (13 groups) with regard to their effect on cardiometabolic health (**S1 Table**). The consumption of beneficially or adversely rated food groups were categorized into quintiles (or a large 0 consumption group and quartiles among consumers). Quintiles of beneficially rated food groups were scored 0 to 4, whereas quintiles of adversely rated food groups were coded in reverse order (4 to 0). Participants were assigned their diet quality score by summing the quintile scores across the 33 food groups, with higher scores indicating better purported health benefit. Neutrally rated food groups did not contribute to the a priori diet quality score. The theoretical maximum value of the score was 132.

### Blood pressure measurement and incidence of hypertension

Systolic and diastolic blood pressures were measured in a quiet room after a 5-minute rest period. Three measurements were taken at 1-min intervals with the participants in seated position; the average of the second and third readings was used in the analyses. At year 10 and 15, random-zero sphygmomanometers were used (Hawksley, WA Baum Co Inc., Copiague, NY), whereas at year 20 blood pressures were measured with automated oscillometers (Omron model HEM907XL; Omron, Mannheim, Germany). Year 20 and year 25 blood pressures were calibrated to random-zero sphygmomanometer values based on information in 903 people whose blood pressure was measured with both devices. Calibrated year 20 and year 25 systolic blood pressure was [3.74 + 0.96 × measured Omron systolic reading], whereas the calibrated year 20 and year 25 diastolic blood pressure was [1.30 + 0.97 × measured Omron diastolic reading]. Hypertension at year 10, 15, 20, and 25 was defined as a systolic reading of ≥140 mmHg, a diastolic reading ≥90 mmHg, or new use of antihypertensive medication. Blood pressure measurements at follow-up made during pregnancy were treated as missing.

### Other measures

Non-dietary correlates were assessed at year 10. Information on age, sex, race, and education (number of years schooling) was collected via self-report using standardized questionnaires. Cigarette smoking and consumption of alcohol were assessed by a structured interview or by self-administered questionnaire. Alcohol intake (ml/day) was estimated from the self-reported frequency of beer, wine, and liquor consumed per week. The use of different vitamin supplements (i.e., of vitamin A, C, E, beta-carotene, and multivitamins) was asked by questionnaire.

Physical activity was queried by an interviewer-based questionnaire, which asks for amount of time spent in 13 exercise and sports activities during the past year [21]. Total physical activity was the sum of intensity times frequency scores, expressed as exercise units.

Anthropometric variables were measured according to standardized protocols. Body weight was measured to the nearest 0.2 kg with participants wearing light clothing. Height was measured to the nearest 0.5 cm without shoes. Body mass index (BMI) was calculated as weight divided by height squared (kg/m^2^). Waist circumference was measured laterally at the point midway between the iliac crest and the lowest lateral portion of the rib cage and anteriorly at the point midway between the xiphoid process of the sternum and the umbilicus.

Diabetes at years 0, 2, 5, 7 and 10 was defined as fasting serum glucose of ≥7.0 mmol/L (126 mg/dL) or the use of anti-diabetic medication [22]. History of diabetes was defined as having diabetes at any of these years.

### Statistical analysis

Cox proportional-hazard regression analysis was used to compute hazard ratios of incident hypertension, occurring between years 10 and 25, according to year 10 plasma ascorbic acid, year 7 a priori diet quality score, and intake of dietary vitamin C and food groups high in vitamin C content. The onset of hypertension at follow-up was estimated at the midpoint between the last exam when the participant was without hypertension and the first exam at which hypertension was noted. People who remained free of hypertension were assigned a follow-up of either 5, 10 or 15 years, depending on when they were seen for the last time since year 10.

In time-dependent Cox regression models, we used year 10 concentrations of ascorbic acid for follow-up between year 10 and year 15, and replaced these values with year 15 ascorbic acid for the period between year 15 and year 25. For participants who were still at risk at year 15, we imputed missing year 15 ascorbic acid by multiplying their year 10 ascorbic acid values with the ratio of the median year 15 ascorbic acid in all available participants to the corresponding median at year 10 [that is, 1.02 × (year 10 ascorbic acid)]. A similar approach was used to update the a priori diet score at year 20, with missing year 20 values being imputed by multiplying their year 7 value by 1.04.

Possible deviations from linearity in the associations were evaluated by fitting fractional polynomials [23]. However, we did not observe worrisome deviations from linearity, so we modeled the plasma ascorbic acid and a priori diet quality score as continuous terms (per 1-SD increment) as well as in quartiles. We first analyzed the associations of hypertension with plasma ascorbic acid and a priori diet quality score separately. We fitted 3 multivariable-adjusted models. Age, sex, race, center, and education were included in the first model. In the second model we further adjusted for smoking, alcohol intake, and physical activity (all at year 10), and, for the a priori diet quality score and consumption of food groups, also for energy intake at year 7. Alcohol was adjusted for because although consumption of alcoholic beverages (beer, liquor, and wine) was rated as beneficial in the a priori diet quality score, alcohol intake is actually a risk factor for hypertension [24]. The third model also adjusted for BMI, waist circumference, history of diabetes, and measured systolic blood pressure (all at year 10). These factors were adjusted for in a separate model because measures of overweight (and consequently diabetes) were considered to be, at least partially, intermediate factors in the causal pathway, whereas systolic blood pressure is part of the outcome of hypertension. To evaluate whether plasma ascorbic acid and a priori diet score were independently related to incident hypertension, we included plasma ascorbic acid and the diet score in the same Cox regression model so as to mutually adjust for their effects.

## Results

### Characteristics of the study population by plasma ascorbic acid (year 10) and *a priori* diet quality score (year 7)

The mean (SD) plasma ascorbic acid at year 10 in 2884 people without hypertension was 48.6 (19.6) μmol/L (to convert values to mg/dL, multiply μmol/L by 0.0176). Of these people, plasma ascorbic acid was on average 4.4 μmol/L lower in 288 people without information on a priori diet quality score at year 7 than in 2596 people with diet quality scores (P value = 0.0003). This was mostly explained by proportionally more blacks and lower educated persons among those with missing diet quality scores (P value = 0.21 after controlling for race and education). The mean diet quality score at year 7 in the sample of 2596 people was 67.8 (SD 12.0). Year 10 plasma ascorbic acid and year 7 diet quality score had similar year 10 correlates (**S2 Table**). Women, whites, and people with more education and of higher age had higher values for both measures than did men, blacks, persons with less education, and those of younger age. They also had more often a lifestyle pattern considered as healthy.

### Association of year 7 *a priori* diet quality score with year 10 plasma ascorbic acid

In the 2596 people with both year 7 a priori diet quality score and year 10 plasma ascorbic acid, plasma ascorbic acid correlated more strongly with the a priori diet quality score (Pearson’s *r* = 0.29) than with intakes of single fruit and vegetable groups. The strongest food group correlations with plasma ascorbic acid were seen for fruit (*r* = 0.18) and dark green vegetables (*r* = 0.16), while the correlation between plasma ascorbic acid and dietary vitamin C was 0.24. In a linear regression model, after adjustment for age, sex, race, center, education, lifestyle, and year 7 energy intake, each 12-point higher a priori diet quality score at year 7 was associated with mean 3.7 μmol/L (95% CI 2.9 to 4.6) higher plasma ascorbic acid at year 10. This value became 3.5 μmol/L (95% CI 2.7 to 4.4) after further adjustment for BMI, waist circumference and history of diabetes. This latter estimate was essentially similar in never-smokers at year 10 and in non-users of vitamin supplements at year 10, but was lower in blacks (2.6 μmol/L, 95% CI 1.2 to 3.9) than in whites (beta 3.9 μmol/L, 95% CI 2.9 to 5.0, with P for interaction by race = 0.0002).

### Incidence of hypertension between year 10 and 25

Of the 2884 people who were followed between years 10 and 25, we identified 840 new cases of hypertension (29.1%). The percentage of people with incident hypertension was 17.2 among white women, 23.2 among white men, 39.1 among black men, and 42.6 among black women. The number of incident cases in the sample of 2596 people with year 7 dietary quality scores was 750 (28.9%).

### Plasma ascorbic acid at year 10 and incident hypertension between years 10 and 25

**Fig 1:**
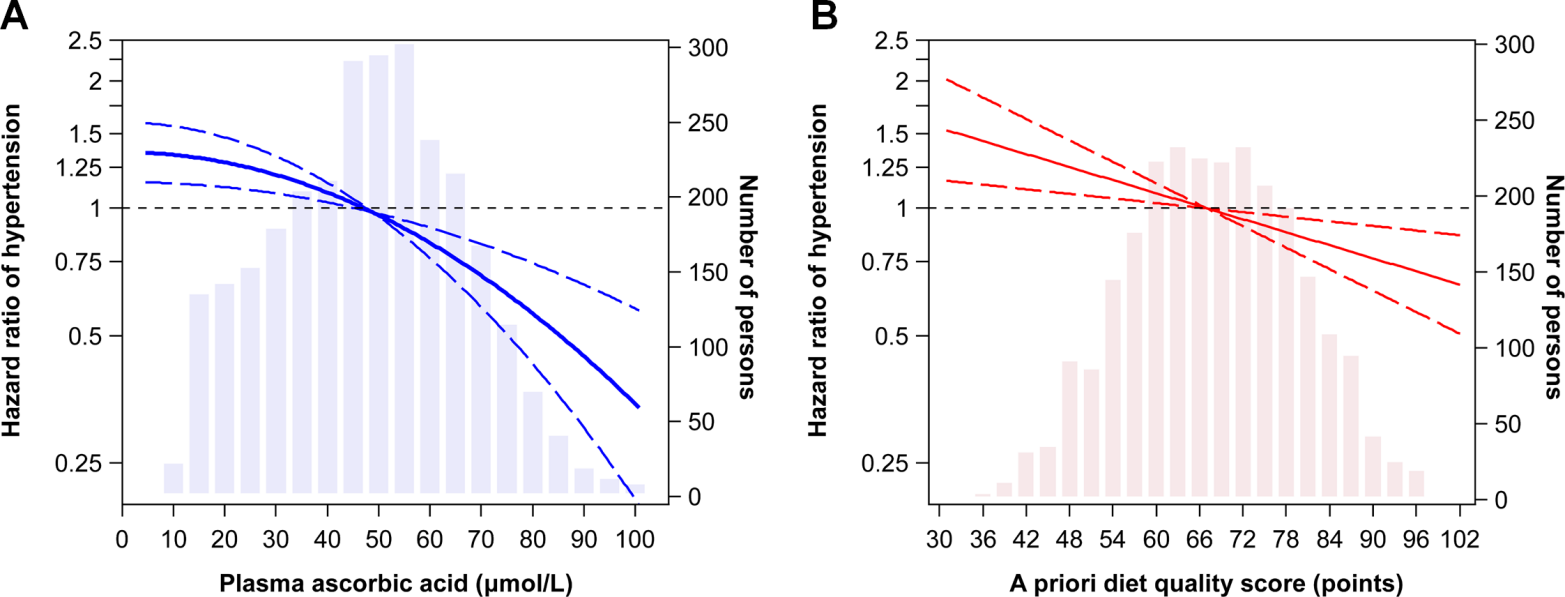
Dose-response associations of plasma ascorbic acid (A) and the diet quality score (B) with incident hypertension. Linearity of the shape of the associations was evaluated with fractional polynomials, with P values for non-linearity being 0.02 for plasma ascorbic acid and 0.79 for the dietary quality score. Adjustments were according to multiple-adjustment model 2.

Plasma ascorbic acid was inversely associated with incident hypertension. For example, the hazard ratio for hypertension for each SD higher plasma ascorbic acid was 0.85 (**Table 1**). Adjustment for BMI, waist circumference, diabetes history, and systolic blood pressure attenuated this hazard ratio to 0.91. When year 10 ascorbic acid values were updated at year 15 in time-dependent analysis, these two hazard ratios (95% CI) were 0.87 (0.82 to 0.93) and 0.92 (0.86 to 0.99), respectively. Across quartiles of year 10 ascorbic acid, there was a suggestion that inverse association became stronger in the higher range of ascorbic acid. The dose-response curve based on the fractional polynomial analysis indicated, however, only a subtle deviation from linearity (**Fig 1A**).

**Table 1:** Hazard ratios for incident hypertension occurring between year 10 and year 25 according to plasma concentrations of ascorbic acid at year 10: Coronary Artery Risk Development in Young Adults (CARDIA) Study ^1^.

	Quartiles of year 10 plasma ascorbic acid				*P* value for linear trend ^2^	Per 19.6 μmol/L increase ^3^
	1 (lowest)	2	3	4 (highest)		
Plasma ascorbic acid (μmol/L), median (range)	24.0 (4.5–34.3)	42.3 (34.4–48.8)	54.8 (48.9–61.8)	70.0 (61.9–211.2)		
No. incident cases/N	274/721	225/720	188/723	153/720		
**Hazard ratio (95% CI):**						
Model 1 ^4^	Reference	0.88 (0.73, 1.05)	0.74 (0.62, 0.90)	0.63 (0.51, 0.78)	<0.0001	0.86 (0.80, 0.92)
Model 2 ^5^	Reference	0.87 (0.72, 1.04)	0.74 (0.60, 0.89)	0.62 (0.50, 0.77)	<0.0001	0.85 (0.79, 0.92)
Model 3 ^6^	Reference	0.88 (0.74, 1.06)	0.80 (0.66, 0.98)	0.72 (0.58, 0.89)	0.001	0.91 (0.84, 0.98)

Abbreviations: CI, confidence interval.

^1^ Shown are hazard ratios for incident hypertension according to year 10 (1995–1996) plasma ascorbic acid in 2884 participants without a history of hypertension at year 10.

^2^ P value for modeling median values for each quartile of plasma ascorbic acid as a continuous variable.

^3^ Hazard ratio per SD higher plasma ascorbic acid.

^4^ Adjusted for age (years), sex, race, center, and education (years).

^5^ Further adjusted for cigarette smoking (dummy variables for current and former cigarette smoking), alcohol intake (ml/day), physical activity score (exercise units), and use of a vitamin supplement (yes/no) (all at year 10).

^6^ Further adjusted for BMI (kg/m^2^), waist circumference (cm), history of diabetes, and systolic blood pressure (mmHg) (all at year 10).

After controlling for demographic and lifestyle factors, the inverse association was stronger in whites (hazard ratio per 1-SD higher year 10 plasma ascorbic acid 0.72, 95% CI 0.64 to 0.82) than in blacks (hazard ratio 0.94, 95% CI 0.85 to 1.04, P value for interaction 0.0001), whereas no difference was seen by sex.

To evaluate any uncontrolled confounding by smoking, we restricted the analysis to never-smokers at year 10 (n = 1758) and found very similar results. The hazard ratio for each SD higher ascorbic acid, after adjustment for demographic and lifestyle factors, was 0.83 (95% CI 0.75 to 0.92). The hazard ratio in people not using a vitamin supplement at year 10 (n = 1562) was 0.86 (95% CI 0.78–0.95).

### *A priori* diet quality score at year 7 and incident hypertension between years 10 and 25

The a priori diet quality score was also inversely associated with incident hypertension (**Table 2**), and this was in a linear fashion (**Fig 1B**). After adjusting for demographic and lifestyle factors, a 12-point (1 SD) higher score corresponded to a hazard ratio of 0.86, which was very similar by sex and race. This hazard ratio became 0.88 after further adjustment for BMI, waist circumference, diabetes history, and systolic blood pressure. These hazard ratios (95% CI) were 0.87 (0.79 to 0.95) and 0.89 (0.81 to 0.98), respectively, in time-dependent analysis in which year 7 diet score values were updated at year 20.

**Table 2:** Hazard ratios for incident hypertension occurring between year 10 and year 25 according to the *a priori* diet quality score at year 7: Coronary Artery Risk Development in Young Adults (CARDIA) Study ^1^.

	Quartiles of year 7 *a priori diet* quality score				*P* value for linear trend ^2^	Per 12 points increase ^3^
	1 (lowest)	2	3	4 (highest)		
Diet quality score, median (range)	54 (31–59)	63 (60–67)	72 (68–76)	82 (77–102)		
No. incident cases/N	251/654	207/629	165/667	127/646		
**Hazard ratio (95% CI):**						
Model 1 ^4^	Reference	0.89 (0.74, 1.08)	0.79 (0.64, 0.98)	0.75 (0.59, 0.96)	0.01	0.89 (0.83, 0.96)
Model 2 ^5^	Reference	0.87 (0.72, 1.05)	0.77 (0.62, 0.95)	0.73 (0.56, 0.94)	0.007	0.88 (0.82, 0.95)
Model 3 ^6^	Reference	0.89 (0.74, 1.08)	0.80 (0.64, 0.99)	0.81 (0.63, 1.04)	0.05	0.90 (0.84, 0.97)

Abbreviations: CI, confidence interval.

^1^ Shown are hazard ratios for incident hypertension occurring between years 10 and 25 by year 7 (1992–1993) diet quality score in 2596 participants without a history of hypertension at year 10.

^2^ P value for modeling median values for each quartile of the diet quality score as a continuous variable.

^3^ Hazard ratio per SD higher diet score.

^4^ Adjusted for age (years), sex, race, center, and education (years).

^5^ Further adjusted for cigarette smoking (dummy variables for current and former cigarette smoking), alcohol intake (ml/day), physical activity score (exercise units), use of a vitamin supplement (yes/no) (all at year 10), and energy intake (kcal/day) at year 7.

^6^ Further adjusted for BMI (kg/m^2^), waist circumference (cm), history of diabetes, and systolic blood pressure (mmHg) (all at year 10).

The association was very similar in 1590 never-smokers at year 10 (hazard ratio per 1-SD higher year 7 diet score adjusted for demographic and lifestyle variables: 0.87, 95% CI 0.76 to 0.98) and in 1386 non-users of vitamin supplements at year 10 (hazard ratio: 0.84, 95% CI 0.74 to 0.95). No effect modification by race or sex was seen.

Neither supplemental vitamin C intake at year 7 (hazard ratio for each 100 mg/day 0.99, 95% CI 0.97 to 1.02) nor use of multivitamin supplements at year 10 (hazard ratio 1.06 for users vs. non-users, 95% CI 0.92 to 1.22) related to incident hypertension after adjustment according to multiple-adjusted model 2. Higher dietary vitamin C, however, was inversely related to hypertension (hazard ratio 0.88, 95% CI 0.81 to 0.96 per 100-mg higher intake, adjusted for model 2 variables). Of eight food groups containing vitamin C, inverse association were seen for dark green vegetables, fruit, fruit juice, and citrus fruit juice (**S3 Table**). Citrus fruit was not related to hypertension, though the range of intake was limited.

### Independent associations of plasma ascorbic acid and *a priori* quality score with incident hypertension

To evaluate whether the associations of plasma ascorbic acid and diet quality score were independent of each other, we fitted one Cox regression model with both variables as linear terms, along with the demographic and lifestyle factors, and energy intake. The hazard ratio (95% CI) for each 19.6-μmol/L higher plasma ascorbic acid was 0.86 (0.79–0.93) and that per 12-points higher diet quality score 0.89 (0.81–0.97). After further adjustment for BMI, waist circumference, diabetes history, and systolic blood pressure, the hazard ratios were virtually identical for both ascorbic acid (hazard ratio 0.91, 95% CI 0.84–0.99) and diet quality score (hazard ratio 0.90, 95% CI 0.82–0.99). Also, there was no evidence of a three-way interaction between plasma ascorbic acid, a priori diet quality score, and race (P value 0.28).

**Fig 2:**
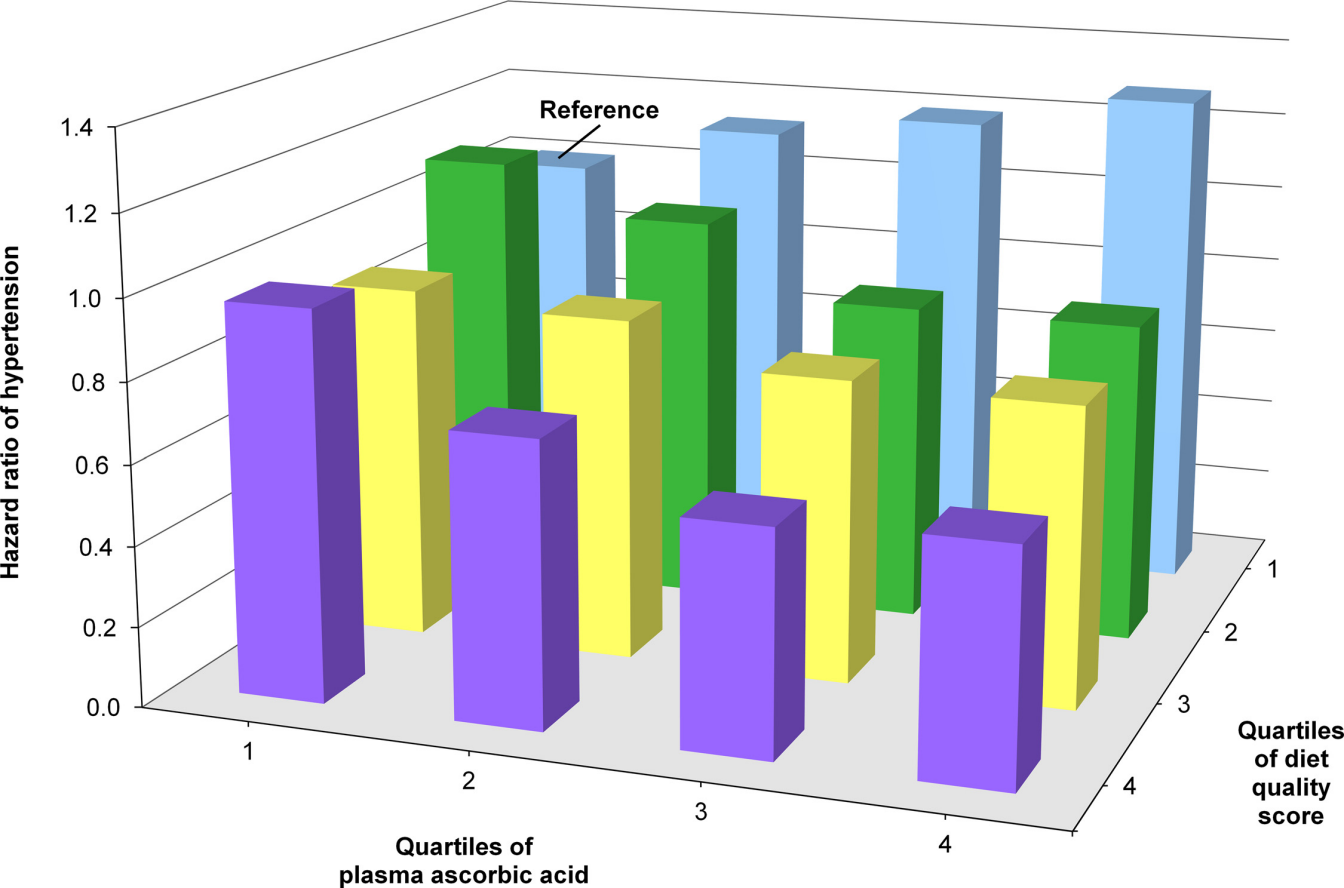
Hazard ratios according to cross-stratified quartiles of plasma ascorbic acid and diet quality score with incident hypertension. People in the lowest quartile of both plasma ascorbic acid and diet quality score served as the reference. Adjustments were according to multivariable-adjustment model 2. There was a suggestion of an interaction between ascorbic acid and diet quality score (P value 0.08).

We finally cross-stratified quartiles of both plasma ascorbic acid and a priori diet quality score to investigate their joint association with incident hypertension. In multiple-adjustment model 2, plasma ascorbic acid was inversely related to hypertension among the three highest quartiles of the a priori diet score (**Fig 2**). In the bottom quartile of the a priori diet score, however, the opposite was seen, that is, higher ascorbic acid tended to be related to higher hypertension risk (P value of product term ascorbic acid times diet quality score 0.08). The a priori diet score appeared to be not related to hypertension in the bottom quartile of plasma ascorbic acid, whereas the inverse association gradually increased across the three remaining higher quartiles.

## Discussion

In this prospective cohort study among community-dwelling US adults, higher values of circulating concentrations of plasma ascorbic acid and the a priori diet quality score related to lower risk of hypertension during 15 years of follow-up. These inverse associations were independent of each other, suggesting that plasma ascorbic acid and diet quality have their own association with risk of hypertension.

Only one previous prospective study assessed plasma ascorbic acid in relation to changes in blood pressure over time and reported an inverse association [17]. Our findings extend that study in that this inverse association persists after adjustment for overall diet quality. One explanation is that plasma ascorbic acid not only marks diet quality, as shown in our data and other data [15], but also other factors that influence blood pressure. These include metabolic factors that may ‘use’ circulating vitamin C, for example in protection against oxidative stress or inflammation. For example, lower plasma ascorbic acid values relate to higher levels of C-reactive protein [25], which, in turn, may contribute to hypertension [26]. We cannot, however, exclude the possibility that plasma ascorbic acid marks other aspects of diet quality not covered in the current a priori diet quality score.

Although we could not clarify whether vitamin C causally contributes to a healthy blood pressure in itself, we did observe that dietary vitamin C and several foods high in vitamin C were inversely related to hypertension, whereas supplemental vitamin C was not. This may suggest that circulating ascorbic acid is preferably improved by consuming foods rich in vitamin C, in addition to not smoking and eating other foods that are low in vitamin C but nevertheless prevent ascorbic acid from depleting.

In the current study, higher a priori diet scores were related to a lower risk of hypertension, which supports the findings of randomized controlled trials on dietary patterns and blood pressure. For example, the DASH trial not only showed that a diet rich in fruit, vegetables and low-fat dairy lowers systolic and diastolic blood pressure, but also that the magnitude of effect was stronger than that of high fruit and vegetable intakes alone [27]. Our finding that the inverse association of the a priori diet quality score was not attenuated after adjustment for plasma ascorbic acid suggests that diet quality favorably affects blood pressure by pathways that do not involve ascorbic acid. For example, such pathways could involve dietary fiber, sodium, potassium, magnesium and calcium, all nutrients which were favorably correlated with the a priori diet quality score in the current study and have been found to affect blood pressure in humans [28–31].

We found that the inverse association of plasma ascorbic acid, but not that of the priori diet score, and risk of hypertension was stronger in whites than in blacks. The relation between the a priori diet score and plasma ascorbic acid was also stronger in whites than in blacks. Taken together, these findings may indicate that plasma ascorbic acid in blacks has different determinants than in whites. Given the lack of significant association between ascorbic acid and hypertension in blacks, these determinants may not necessarily be risk factors of high blood pressure. This merits further research.

After cross-stratification of plasma ascorbic acid and a priori diet quality score, higher plasma ascorbic acid tended to related with higher hypertension risk in those with diets of poor quartile (bottom quartile), whereas inverse associations were seen in the three higher quartiles of the diet score. The diet score itself was not related to hypertension in those with the lowest plasma ascorbic acid levels, whereas an inverse association gradually appeared across the remaining increasing quartiles of plasma ascorbic acid. However, in view of our lack of understanding of this complex interplay and the lack of statistically significant interaction, we consider this finding as “hypothesis generating.”

One limitation of our study is that for the analysis of the a priori diet score, we used dietary intake assessed at year 7, that is, the time point closest to the measurement of plasma ascorbic acid (year 10). As a consequence, there was a 3-year lag-time between the a priori diet score and plasma ascorbic acid. However, because the diet score has been found to track highly within people over a period of 20 years [19], we do not expect this to substantially influence our findings. Another limitation is that our primary findings on plasma ascorbic acid rely on a single measurement in each participant. This will likely underestimate the association between ascorbic acid and hypertension because of misclassification associated with a single measurement [32]. However, because results of using an additional measurement of plasma ascorbic acid taken 5 years after the baseline measurement were similar as our primary findings, this limitation may be of less concern in our study.

## Conclusions

In conclusion, in community-dwelling US adults, higher values of both plasma ascorbic acid and a priori diet quality score independently relate to lower risk of hypertension. This suggests that, apart from being a marker of a healthy diet, plasma ascorbic acid may at least partly favorably influence blood pressure in itself, or mark something else that influences blood pressure, in addition to the dietary aspects assessed in the a priori diet quality score. Given the inverse associations seen for dietary vitamin C and several foods rich in vitamin C, but not for supplemental vitamin C, it seems that ascorbic acid status may best be improved by increasing dietary sources of vitamin C, in addition to not smoking and any other dietary sources low in vitamin C that prevent ascorbic acid from depletion.

## Supporting Information

S1 Table Year 10 characteristics by plasma ascorbic acid at year 10 and the *a priori* diet quality score at year 7: Coronary Artery Risk Development in Young Adults (CARDIA) Study (DOCX)

S2 Table Hazard ratios for incident hypertension occurring between year 10 and year 25 according to consumption of food sources of vitamin C at year 7: Coronary Artery Risk Development in Young Adults (CARDIA) Study (DOCX)

S3 Table Hazard ratios for incident hypertension occurring between year 10 and year 25 according to consumption of food sources of vitamin C at year 7: Coronary Artery Risk Development in Young Adults (CARDIA) Study (DOCX)

## Abbreviations

BMI: body mass index
CARDIA: Coronary Artery Risk Development in Young Adults
CI: confidence interval
