# Supplementary material for: Plasma Ascorbic Acid, A Priori Diet Quality Score, and Incident Hypertension: A Prospective Cohort Study
Source: PLoS One. 2015 Dec 18;10(12):e0144920. doi: 10.1371/journal.pone.0144920 (PMC4684305; doi:10.1371/journal.pone.0144920)
Supplement: S1 Table — (DOCX) [file pone.0144920.s001.docx]

**S1 Table.** Ratings of food groups for construction of the a priori diet quality score in the Coronary Artery Risk Development in Young Adults (CARDIA) Study

| Food group | Beneficially rated | Adversely rated | Neutrally rated |
| --- | --- | --- | --- |
| Avocado | X |  |  |
| Beans | X |  |  |
| Beer | X |  |  |
| Coffee | X |  |  |
| Fatty fish | X |  |  |
| Fruit | X |  |  |
| Green vegetables | X |  |  |
| Lean fish | X |  |  |
| Low-fat dairy | X |  |  |
| Liquor | X |  |  |
| Oil | X |  |  |
| Other vegetables | X |  |  |
| Poultry | X |  |  |
| Seed and nuts | X |  |  |
| Soy products | X |  |  |
| Tea | X |  |  |
| Tomato | X |  |  |
| Whole grains | X |  |  |
| Wine | X |  |  |
| Yellow vegetables | X |  |  |
| Butter |  | X |  |
| Fried poultry and fish |  | X |  |
| Fried potato |  | X |  |
| Grain dessert |  | X |  |
| Organ meat |  | X |  |
| Processed meat |  | X |  |
| Regular red meat |  | X |  |
| Salty snacks |  | X |  |
| Sauces |  | X |  |
| Soft drinks |  | X |  |
| Sweet breads |  | X |  |
| Sweet extra’s |  | X |  |
| Whole-fat dairy |  | X |  |
| Chocolate |  |  | X |
| Diet soft drinks |  |  | X |
| Eggs |  |  | X |
| Fruit juice |  |  | X |
| Lean red meat |  |  | X |
| Margarine |  |  | X |
| Meal replacements |  |  | X |
| Pickled food |  |  | X |
| Potatoes |  |  | X |
| Refined grains |  |  | X |
| Shellfish |  |  | X |
| Soups |  |  | X |
| Sugar substitutes |  |  | X |
