# Supplementary material for: Plasma Ascorbic Acid, A Priori Diet Quality Score, and Incident Hypertension: A Prospective Cohort Study
Source: PLoS One. 2015 Dec 18;10(12):e0144920. doi: 10.1371/journal.pone.0144920 (PMC4684305; doi:10.1371/journal.pone.0144920)
Supplement: S2 Table — (DOCX) [file pone.0144920.s002.docx]

**S2 Table.** Year 10 characteristics by plasma ascorbic acid at year 10 and the a priori diet quality score at year 7: Coronary Artery Risk Development in Young Adults (CARDIA) Study ^1^

|  | Quartiles of year 10 plasma ascorbic acid | | | |  | Quartiles of year 7 a priori diet quality score | | | |
| --- | --- | --- | --- | --- | --- | --- | --- | --- | --- |
| Year 10 characteristic | 1 (n=721) | 2 (n=720) | 3 (n=723) | 4 (n=720) |  | 1 (n=654) | 2 (n=629) | 3 (n=667) | 4 (n=646) |
| Plasma ascorbic acid (µmol/L) ^2^ | 23.7 | 42.3 | 54.8 | 70.0 |  | 7.4 | 8.2 | 8.9 | 10.1 |
| Diet quality score at year 7 | 62.8 | 66.7 | 69.5 | 71.8 |  | 52.4 | 63.5 | 72.0 | 83.2 |
| Mean age (years) | 34.7 | 34.9 | 35.0 | 35.2 |  | 33.9 | 34.8 | 35.6 | 35.9 |
| Sex (% female) | 50.8 | 50.3 | 55.9 | 68.2 |  | 52.0 | 52.5 | 57.1 | 64.2 |
| Race (% black) | 52.2 | 47.1 | 42.2 | 31.4 |  | 66.7 | 50.2 | 30.4 | 14.7 |
| Education (years) | 14.9 | 15.6 | 16.2 | 16.4 |  | 14.7 | 15.5 | 16.3 | 16.9 |
| Mean body mass index (kg/m^2^) | 28.0 | 28.0 | 26.2 | 25.5 |  | 28.3 | 27.3 | 26.6 | 25.3 |
| Mean height (cm) | 171.0 | 171.4 | 171.5 | 169.3 |  | 170.9 | 171.1 | 170.8 | 170.5 |
| *Mean waist circumference (cm)* |  |  |  |  |  |  |  |  |  |
| Men | 90.9 | 90.8 | 88.4 | 85.8 |  | 90.6 | 89.4 | 89.6 | 87.8 |
| Women | 85.3 | 83.5 | 79.1 | 77.4 |  | 85.6 | 82.2 | 80.0 | 76.3 |
| *Smoking status (%)* |  |  |  |  |  |  |  |  |  |
| Current ≥15 cigarettes/day | 19.7 | 7.9 | 4.7 | 4.9 |  | 10.7 | 10.7 | 7.7 | 6.2 |
| Current <15 cigarettes/day | 19.0 | 14.2 | 10.9 | 7.4 |  | 15.3 | 14.0 | 10.9 | 9.1 |
| Former | 11.2 | 16.8 | 17.2 | 22.4 |  | 13.5 | 13.5 | 18.6 | 24.9 |
| Never | 50.1 | 61.1 | 67.2 | 65.4 |  | 60.6 | 61.8 | 62.8 | 59.8 |
| Median alcohol intake (ml/day) | 2.7 | 2.4 | 2.4 | 2.4 |  | 0.0 | 0.0 | 4.8 | 7.2 |
| Mean physical activity (exercise units) | 305 | 329 | 362 | 353 |  | 278 | 318 | 345 | 403 |
| History of diabetes (%) | 2.9 | 2.4 | 1.9 | 0.7 |  | 2.6 | 1.9 | 2.1 | 1.1 |

**S2 Table.** Cont.

|  | Quartiles of year 10 plasma ascorbic acid | | | |  | Quartiles of year 7 a priori diet quality score | | | |
| --- | --- | --- | --- | --- | --- | --- | --- | --- | --- |
| Year 10 characteristic | 1 (n=721) | 2 (n=720) | 3 (n=723) | 4 (n=720) |  | 1 (n=654) | 2 (n=629) | 3 (n=667) | 4 (n=646) |
| Use of vitamin supplements (%) ^3^ | 27.7 | 42.1 | 53.1 | 60.4 |  | 33.8 | 44.4 | 49.3 | 59.0 |
| Use of vitamin C supplements at y 7 (%) | 19.4 | 32.9 | 39.2 | 46.1 |  | 23.4 | 31.2 | 38.2 | 45.7 |
| Median intake vitamin C from supplements in users at y 7 (mg/d) ^4^ | 60.0 | 60.0 | 65.7 | 100.0 |  | 60.0 | 60.0 | 65.7 | 105.9 |
| *Mean intake of food groups at y 7* |  |  |  |  |  |  |  |  |  |
| Dark green vegetables (serv./d) | 0.38 | 0.49 | 0.59 | 0.65 |  | 0.25 | 0.38 | 0.53 | 0.96 |
| Yellow vegetables (serv./d) | 0.17 | 0.23 | 0.27 | 0.31 |  | 0.11 | 0.18 | 0.26 | 0.44 |
| Tomato (serv./d) | 0.56 | 0.59 | 0.68 | 0.72 |  | 0.44 | 0.55 | 0.66 | 0.91 |
| Other vegetables (serv./d) | 2.00 | 2.17 | 2.30 | 2.37 |  | 1.55 | 1.90 | 2.25 | 3.15 |
| Fruit (serv./d) | 1.09 | 1.59 | 1.82 | 1.90 |  | 0.94 | 1.39 | 1.68 | 2.41 |
| Of which citrus fruit (serv./d) | 0.14 | 0.21 | 0.24 | 0.23 |  | 0.12 | 0.19 | 0.22 | 0.29 |
| Fruit juice (serv./d) | 1.00 | 1.36 | 1.63 | 1.52 |  | 1.21 | 1.38 | 1.45 | 1.48 |
| Of which citrus fruit juice | 0.57 | 0.79 | 0.94 | 0.85 |  | 0.67 | 0.73 | 0.89 | 0.87 |
| *Mean nutrient intake at y 7* |  |  |  |  |  |  |  |  |  |
| Vitamin C (mg/d) ^4^ | 137.2 | 167.1 | 185.7 | 178.0 |  | 133.4 | 150.6 | 165.4 | 202.3 |
| Sodium (mg/d) ^4^ | 4383 | 4314 | 4289 | 3960 |  | 4417 | 4263 | 4080 | 4176 |
| Potassium (mg/d) ^4^ | 3450 | 3701 | 3846 | 3758 |  | 3182 | 3520 | 3728 | 4334 |
| Magnesium (mg/d) ^4^ | 378 | 397 | 413 | 402 |  | 343 | 379 | 403 | 466 |
| Calcium (mg/d) ^4^ | 1053 | 1106 | 1122 | 1090 |  | 1016 | 1092 | 1094 | 1171 |
| Dietary fiber (g/d) ^4^ | 20.4 | 22.4 | 23.8 | 24.9 |  | 18.3 | 20.8 | 23.8 | 28.8 |

^1^ Shown are characteristics at year 10, unless otherwise indicated, according to plasma ascorbic acid at year 10 (1995-1996) and diet quality score at year 7 (1992-1993) in participants without a history of hypertension at year 10.

^2^ To convert ascorbic acid values to mg/dL, multiply µmol/L by 0.0176.

^3^ Vitamin A, vitamin C, vitamin E, beta-carotene, or a multivitamin supplement at year 10.

^4^ Based on n=907 for the analysis by quartiles of plasma ascorbic acid and n=899 for that by quartiles of the diet quality score. Sodium intake does not include salt added in cooking or at table
