# Supplementary material for: Plasma Ascorbic Acid, A Priori Diet Quality Score, and Incident Hypertension: A Prospective Cohort Study
Source: PLoS One. 2015 Dec 18;10(12):e0144920. doi: 10.1371/journal.pone.0144920 (PMC4684305; doi:10.1371/journal.pone.0144920)
Supplement: S3 Table — (DOCX) [file pone.0144920.s003.docx]

**S3 Table.** Hazard ratios for incident hypertension occurring between year 10 and year 25 according to consumption of food sources of vitamin C at year 7: Coronary Artery Risk Development in Young Adults (CARDIA) Study ^1^

|  | Quartiles of foods rich in vitamin C | | | | *P* value  for linear  trend ^2^ | Per serving |
| --- | --- | --- | --- | --- | --- | --- |
|  | 1 (lowest) | 2 | 3 | 4 (highest) |  |  |
| *Dark green vegetables* |  |  |  |  |  |  |
| Median intake (servings/d) | 0.02 (0, 0.10) ^3^ | 0.20 (0.11, 0.31) | 0.46 (0.32, 0.69) | 1.15 (0.70, 8.5) |  |  |
| No. incident cases/N | 217/660 | 207/655 | 189/648 | 137/633 |  |  |
| Hazard ratio (95% CI) |  |  |  |  |  |  |
| Model 1 ^4^ | Reference | 0.91 (0.75, 1.10) | 0.91 (0.75, 1.11) | 0.72 (0.57, 0.89) | 0.004 | 0.86 (0.76, 0.98) |
| Model 2 ^5^ | Reference | 0.91 (0.75, 1.10) | 0.92 (0.75, 1.12) | 0.73 (0.58, 0.92) | 0.01 | 0.88 (0.77, 1.00) |
| Model 3 ^6^ | Reference | 0.95 (0.78, 1.15) | 0.92 (0.75, 1.13) | 0.77 (0.61, 0.97) | 0.02 | 0.87 (0.76, 0.99) |
| *Yellow vegetables* |  |  |  |  |  |  |
| Intake (servings/d) | 0 (0, 0.02) | 0.07 (0.03, 0.12) | 0.19 (0.13, 0.29) | 0.51 (0.30, 6.6) |  |  |
| No. incident cases/N | 213/628 | 199/663 | 182/658 | 156/647 |  |  |
| Hazard ratio (95% CI) |  |  |  |  |  |  |
| Model 1 ^4^ | Reference | 0.97 (0.80, 1.17) | 0.97 (0.80, 1.19) | 0.85 (0.68, 1.04) | 0.11 | 0.83 (0.67, 1.02) |
| Model 2 ^5^ | Reference | 0.98 (0.80, 1.19) | 0.99 (0.81, 1.21) | 0.88 (0.71, 1.09) | 0.24 | 0.86 (0.69, 1.07) |
| Model 3 ^6^ | Reference | 1.04 (0.85, 1.27) | 1.10 (0.90, 1.36) | 0.97 (0.78, 1.22) | 0.70 | 0.90 (0.74, 1.09) |

**S3 Table.** Cont.

|  | Quartiles of foods rich in vitamin C | | | | *P* value  for linear  trend ^2^ | Per serving |
| --- | --- | --- | --- | --- | --- | --- |
|  | 1 (lowest) | 2 | 3 | 4 (highest) |  |  |
| *Tomato* |  |  |  |  |  |  |
| Intake (servings/d) | 0.16 (0, 0.26) | 0.37 (0.27, 0.48) | 0.64 (0.49, 0.84) | 1.20 (0.85, 9.3) |  |  |
| No. incident cases/N | 238/667 | 197/634 | 175/666 | 140/629 |  |  |
| Hazard ratio (95% CI) |  |  |  |  |  |  |
| Model 1 ^4^ | Reference | 1.10 (0.91, 1.33) | 1.03 (0.84, 1.26) | 0.93 (0.74, 1.16) | 0.36 | 0.90 (0.78, 1.04) |
| Model 2 ^5^ | Reference | 1.12 (0.92, 1.36) | 1.07 (0.87, 1.33) | 0.99 (0.77, 1.27) | 0.74 | 0.93 (0.79, 1.09) |
| Model 3 ^6^ | Reference | 1.13 (0.93, 1.37) | 1.06 (0.86, 1.31) | 0.89 (0.69, 1.14) | 0.23 | 0.89 (0.76, 1.05) |
| *Other vegetables* |  |  |  |  |  |  |
| Intake (servings/d) | 0.75 (0.02, 1.10) | 1.42 (1.10, 1.74) | 2.21 (1.75, 2.84) | 3.9 (2.9, 17.4) |  |  |
| No. incident cases/N | 203/653 | 193/658 | 198/653 | 156/632 |  |  |
| Hazard ratio (95% CI) |  |  |  |  |  |  |
| Model 1 ^4^ | Reference | 1.07 (0.87, 1.30) | 1.20 (0.99, 1.47) | 0.99 (0.80, 1.22) | 0.90 | 0.98 (0.94, 1.03) |
| Model 2 ^5^ | Reference | 1.11 (0.90, 1.36) | 1.28 (1.03, 1.59) | 1.09 (0.86, 1.40) | 0.54 | 1.00 (0.95, 1.05) |
| Model 3 ^6^ | Reference | 1.05 (0.85, 1.29) | 0.94 (0.94, 1.45) | 0.90 (0.70, 1.15) | 0.30 | 0.95 (0.90, 1.00) |

**S3 Table.** Cont.

|  | Quartiles of foods rich in vitamin C | | | | *P* value  for linear  trend ^2^ | Per serving |
| --- | --- | --- | --- | --- | --- | --- |
|  | 1 (lowest) | 2 | 3 | 4 (highest) |  |  |
| *Fruit* |  |  |  |  |  |  |
| Intake (servings/d) | 0.25 (0, 0.54) | 0.82 (0.55, 1.15) | 1.55 (1.15, 2.10) | 3.10 (2.11, 19.6) |  |  |
| No. incident cases/N | 235/653 | 192/652 | 167/648 | 156/643 |  |  |
| HR (95% CI) |  |  |  |  |  |  |
| Model 1 ^4^ | Reference | 0.95 (0.78, 1.15) | 0.81 (0.66, 0.99) | 0.75 (0.61, 0.93) | 0.004 | 0.94 (0.89, 0.98) |
| Model 2 ^5^ | Reference | 0.96 (0.79, 1.17) | 0.82 (0.67, 1.02) | 0.78 (0.63, 0.98) | 0.02 | 0.95 (0.90, 1.00) |
| Model 3 ^6^ | Reference | 0.94 (0.77, 1.14) | 0.88 (0.71, 1.08) | 0.78 (0.62, 0.97) | 0.02 | 0.95 (0.90, 1.00) |
| *Citrus fruit* |  |  |  |  |  |  |
| Intake (servings/d) | 0 (0, 0) | 0.07 (0.01, 0.13) | 0.21 (0.14, 0.32) | 0.57 (0.33, 6.00) |  |  |
| No. incident cases/N | 340/1163 | 150/452 | 121/482 | 139/499 |  |  |
| HR (95% CI) |  |  |  |  |  |  |
| Model 1 ^4^ | Reference | 1.16 (0.96, 1.41) | 0.88 (0.72, 1.09) | 1.00 (0.82, 1.21) | 0.62 | 1.06 (0.89, 1.26) |
| Model 2 ^5^ | Reference | 1.15 (0.95, 1.40) | 0.88 (0.72, 1.09) | 1.03 (0.84, 1.26) | 0.71 | 1.09 (0.92, 1.31) |
| Model 3 ^6^ | Reference | 1.18 (0.97, 1.43) | 0.89 (0.72, 1.10) | 0.95 (0.78, 1.17) | 0.18 | 1.00 (0.84, 1.20) |

**S3 Table.** Cont.

|  | Quartiles of foods rich in vitamin C | | | | *P* value  for linear  trend ^2^ | Per serving |
| --- | --- | --- | --- | --- | --- | --- |
|  | 1 (lowest) | 2 | 3 | 4 (highest) |  |  |
| *Fruit juice* |  |  |  |  |  |  |
| Intake (servings/d) | 0.1 (0, 0.3) | 0.6 (0.3, 0.9) | 1.3 (0.9, 1.9) | 2.9 (1.9, 20.8) |  |  |
| No. incident cases/N | 198/652 | 217/652 | 174/657 | 161/635 |  |  |
| HR (95% CI) |  |  |  |  |  |  |
| Model 1 ^4^ | Reference | 1.12 (0.93, 1.37) | 0.83 (0.67, 1.02) | 0.78 (0.63, 0.97) | 0.002 | 0.96 (0.91, 1.00) |
| Model 2 ^5^ | Reference | 1.14 (0.94, 1.38) | 0.84 (0.68, 1.04) | 0.81 (0.65, 1.01) | 0.008 | 0.96 (0.92, 1.01) |
| Model 3 ^6^ | Reference | 1.06 (0.87, 1.29) | 0.86 (0.70, 1.06) | 0.75 (0.60, 0.94) | 0.002 | 0.95 (0.90, 0.99) |
| *Citrus fruit juice* |  |  |  |  |  |  |
| Intake (servings/d) | 0 (0, 0.06) | 0.22 (0.07, 0.42) | 0.67 (0.43, 1.13) | 1.90 (1.14, 16.0) |  |  |
| No. incident cases/N | 212/646 | 189/642 | 184/662 | 165/646 |  |  |
| HR (95% CI) |  |  |  |  |  |  |
| Model 1 ^4^ | Reference | 0.94 (0.76, 1.14) | 0.87 (0.71, 1.06) | 0.78 (0.64, 0.96) | 0.02 | 0.95 (0.88, 1.02) |
| Model 2 ^5^ | Reference | 0.93 (0.76, 1.13) | 0.87 (0.72, 1.07) | 0.80 (0.65, 0.99) | 0.04 | 0.96 (0.89, 1.03) |
| Model 3 ^6^ | Reference | 0.94 (0.77, 1.15) | 0.82 (0.67, 1.00) | 0.81 (0.65, 0.99) | 0.04 | 0.94 (0.88, 1.01) |

Abbreviations: CI, confidence interval.

^1^ Shown are hazard ratios for incident hypertension occurring between year 10 and year 25 by year 7 (1992-1993) intake of food groups rich in vitamin C in 2596 participants without a history of hypertension at year 10.

^2^ P value for modeling median intake values for each quartile as a continuous variable.

^3^ Median (range) for all such values.

^4^ Adjusted for age (years), sex, race, center, and education (years).

^5^ Further adjusted for cigarette smoking (dummy variables for current and former cigarette smoking), alcohol intake (ml/day), physical activity score (exercise units), use of a vitamin supplement (yes/no) (all at year 10), and energy intake (kcal/day) at year 7.

^6^ Further adjusted for BMI (kg/m^2^), waist circumference (cm), history of diabetes, and systolic blood pressure (mmHg) (all at year 10).
